# Therapeutic Effect of Donepezil on Neuroinflammation and Cognitive Impairment after Moderate Traumatic Brain Injury

**DOI:** 10.3390/life14070839

**Published:** 2024-07-01

**Authors:** Dong Hyuk Youn, Younghyurk Lee, Sung Woo Han, Jong-Tae Kim, Harry Jung, Gui Seung Han, Jung In Yoon, Jae Jun Lee, Jin Pyeong Jeon

**Affiliations:** 1 Institute of New Frontier Research Team, Hallym University College of Medicine, Chuncheon 24252, Republic of Korea; zk61326@naver.com (D.H.Y.); younghyurklee@gmail.com (Y.L.); hsw4070@naver.com (S.W.H.); jtfreejuly@gmail.com (J.-T.K.); harry_88@naver.com (H.J.); 2 Life Genomics Co., Ltd., Research & Development Center, Suwon 16417, Republic of Korea; gshan@lifegenomics.co.kr; 3 Department of Biological Sciences and Bioengineering, Inha University, Incheon 22212, Republic of Korea; yoon3397@gmail.com; 4 Department of Anesthesiology and Pain Medicine, Chuncheon Sacred Heart Hospital, Hallym University College of Medicine, Chuncheon 24252, Republic of Korea; 5 Department of Neurosurgery, Hallym University College of Medicine, Chuncheon 24253, Republic of Korea

**Keywords:** donepezil, traumatic brain injury, cognition, neuroinflammation, mitochondria

## Abstract

Background: Despite the important clinical issue of cognitive impairment after moderate traumatic brain injury (TBI), there is currently no suitable treatment. Here, we used in vitro and in vivo models to investigate the effect of Donepezil—an acetylcholinesterase (AChE) inhibitor—on cognitive impairment in the acute period following injury, while focusing on neuroinflammation and autophagy- and mitophagy-related markers. Methods: The purpose of the in vitro study was to investigate potential neuroprotective effects in TBI-induced cells after donepezil treatment, and the in vivo study, the purpose was to investigate therapeutic effects on cognitive impairment in the acute period after injury by analyzing neuroinflammation and autophagy- and mitophagy-related markers. The in vitro TBI model involved injuring SH-SY5Y cells using a cell-injury controller and then investigating the effect of donepezil at a concentration of 80 μM. The in vivo TBI model was made using a stereotaxic impactor for male C57BL/6J mice. Immuno-histochemical markers and cognitive functions were compared after 7 days of donepezil treatment (1 mg/kg/day). Mice were divided into four groups: sham operation with saline treatment, sham operation with donepezil treatment, TBI with saline treatment, and TBI with donepezil treatment (18 mice in each group). Donepezil treatment was administered within 4 h post-TBI. Results: In vitro, donepezil was found to lead to increased cell viability and 5,5′,6,6′-tetrachloro-1,1′,3,3′-tetraethylbenzimi-dazolylcarbocyanine iodide (JC-1), along with decreased reactive oxygen species (ROS), lactate-dehydrogenase (LDH), 2′-7′-dichlorodihydrofluorescein diacetate (DCFH-DA)-positive cells, and terminal deoxynucleotidyl transferase dUTP nick end labeling (TUNEL)-positive cells. The mRNA and protein expressions of neuroinflammation (Cyclooxygenase-2, COX-2; NOD-like receptor protein 3, NLRP3; Caspase-1; and Interleukin-1 beta, IL-1β), as well as autophagy- and mitophagy-related markers (death-associated protein kinase 1, DAPK1; PTEN-induced kinase 1, PINK1; BCL2/adenovirus E1B 19 kDa protein-interacting protein 3-like, BNIP3L; Beclin-1, BECN1; BCL2-associated X protein, BAX; microtubule-associated protein 1A/1B-light chain 3B (LC3B); Sequestosome-1; and p62) were all found to decrease after donepezil treatment. The in vivo study also showed that donepezil treatment resulted in decreased levels of cortical tissue losses and brain swelling in TBI compared to the TBI group without donepezil treatment. Donepezil treatment was also shown to decrease the mRNA and Western blotting expressions of all markers, and especially COX-2 and BNIP3L, which showed the most significant decreases. Moreover, TBI mice showed an decreased escape latency, increased alteration rate, and improved preference index, altogether pointing to better cognitive performance after donepezil treatment. Conclusions: Donepezil treatment may be beneficial in improving cognitive impairment in the early phase of moderate traumatic brain injury by ameliorating neuroinflammation, as well as autophagy and mitophagy.

## 1. Introduction

Donepezil is a well-known medication for Alzheimer’s disease (AD). It acts as an acetylcholinesterase (AChE) inhibitor, thus compensating for the cholinergic deficiency in the transmission [1,2]. In addition to the role it plays as an AChE inhibitor, donepezil has been reported to improve cognitive impairment via the following mechanisms: First, donepezil reduces mitochondrial amyloid-β (Aβ) accumulation and mitochondrial dysfunction [3]. It also exhibits a beneficial effect on neurometabolism via AMP-activated protein kinase (AMPK)/PGC-1α signaling. Treating neuroblastoma cells with donepezil has been shown to increase mitochondrial biogenesis, ATP levels, and PGC-1α expression. In vitro studies showed that treating neuroblastoma cells with donezepil increases mitochondrial biogenesis, ATP levels, and PCG-1alpha expression [2]. The expressions of IL-1beta and cyclooxygenase-2 enzyme (COX-2), as well as the expression of of c-Jun N-terminal kinase (JNK), were found to decrease in the brain after donepezil treatment, suggesting the efficacy of donepezil in post-traumatic cognitive impairment by alleviating neuroinflammation and tau pathology [4]. Donepezil has also been shown to inhibit α-synuclein (αSyn) aggregation in the olfactory bulb with enhanced autophagy [5,6]. Despite the various therapeutic mechanisms that could be at play, the efficacy of donepezil treatment in post-trauma brain injury (TBI) cognitive impairment in real clinical circumstances remains inconclusive due to mixed study results.

Previous moderate-to-severe TBI studies on cognitive impairment were mainly performed in the subacute or chronic phase after brain injury. More specifically, 49% of patients experienced memory deficits, and 54% had attention deficits in the subacute phase, while the percentage of memory deficits was 21%, and attention deficits was 50% in the chronic phase [7]. TBI can lead to a damaged cholinergic system either through direct injury of cholinergic projections or progressive neuronal loss of specific brain lesions, such as the cholinergic nucleus of the diagonal band of Broca and the medial septal nucleus [8]. Donepezil has been tested to assess whether it can enhance cholinergic neurotransmission, which, in turn, is expected to lead to better cognitive function after TBI. Khateb et al. [9]. reported that 8 out of 10 patients with chronic TBI exhibited significant improvements in both learning and speed of processing after donepezil treatment. In another study, TBI survivors who had experienced TBI at least 1.5 years prior showed significant improvement in immediate and delayed memory portions in the Brief Visual Memory Test—Revised [10]. In a study involving temporally regulated ablation of injury-induced neurogenesis, donepezil was shown to result in improved cognitive function with enhanced neurogenesis in nestin-HSV transgenic mice with TBI [11]. Nevertheless, it is still unclear when and how donepezil treatment should be administered to ameliorate cognitive impairment after brain injury based on previous findings. Also, there was a lack of research on what TBI severity that donepezil showed an effective potential for [12]. Walker et al. [13] reported that donepezil did not lead to a meaningful difference in cognitive impairment in TBI patients with moderate-to-severe TBI severity. We believe that moderate TBI should be considered a separate disease category from both severe TBI and mild TBI [14]. Moderate TBI patients typically do not undergo surgery compared to those with severe TBI, although they show more abnormalities on initial computed tomography than those with mild TBI [14]. When treating actual patients, moderate TBI patients often complain of cognitive impairment from the acute stage. By contrast, severe TBI patients often have difficulty communicating, which can make it difficult to assess cognitive function. Few studies have attempted to assess the effect of donepezil on cognitive impairment in moderate TBI while accounting for clinical characteristics based on TBI severity.

Non-excitatory amino acids (NEAAs) play a crucial role in the pathogenesis of TBI. Specifically, NEAAs such as L-alanine, L-glutamine, glycine, L-histidine, L-serine, taurine, and L-threonine can accelerate synaptic transmission suppression and cause neuronal membrane depolarization during hypoxia, leading to neuronal damage [15]. These amino acids can adversely affect synaptic transmission depending on their concentration and combination, contributing to cognitive decline after TBI. System N and the ala-nine-serine-cysteine transporter 2 (ASCT2) are involved in the harmful effects of NEAAs during hypoxia, making them potential therapeutic targets for TBI treatment. Therefore, it is essential to explore the impact of donepezil on these NEAA-related pathways to mitigate early cognitive impairment post-TBI.

Neuroinflammation and autophagic flux have both been shown to increase immediately in response to brain injury [16]. Recent studies have shown interactions between neuroinflammation and autophagy or mitophagy in the development of Alzheimer’s disease (AD), although there are various pathogenic factors that may affect cognitive impairment [17,18]. The continuous activation of neuroinflammation markers such as NOD-, LRR, pyrin domain-containing protein 3 (NLRP3), and high-mobility group box 1 (HMGB1) has been shown to be associated with cognitive impairment in NLRP3-knockout mice after TBI [19]. Autophagy and mitophagy induced by TBI contribute to the imbalance between autophagosome formation and autophagic flux that possibly interferes with the removal of damaged cells [16]. It is theoretically possible to reduce post-TBI cognitive impairment by relieving neuroinflammation, as well as abnormal autophagy and mitophagy, in the early post-injury period. In this study, we investigated the potential effect of donepezil on cognitive impairment that developed in the early phase following TBI in both in vitro and in vivo models of moderate TBI, while focusing on changes in neuroinflammation, as well as autophagy- and mitophagy-related markers.

## 2. Methods

### 2.1. In Vitro Model of TBI

**Figure 1:**
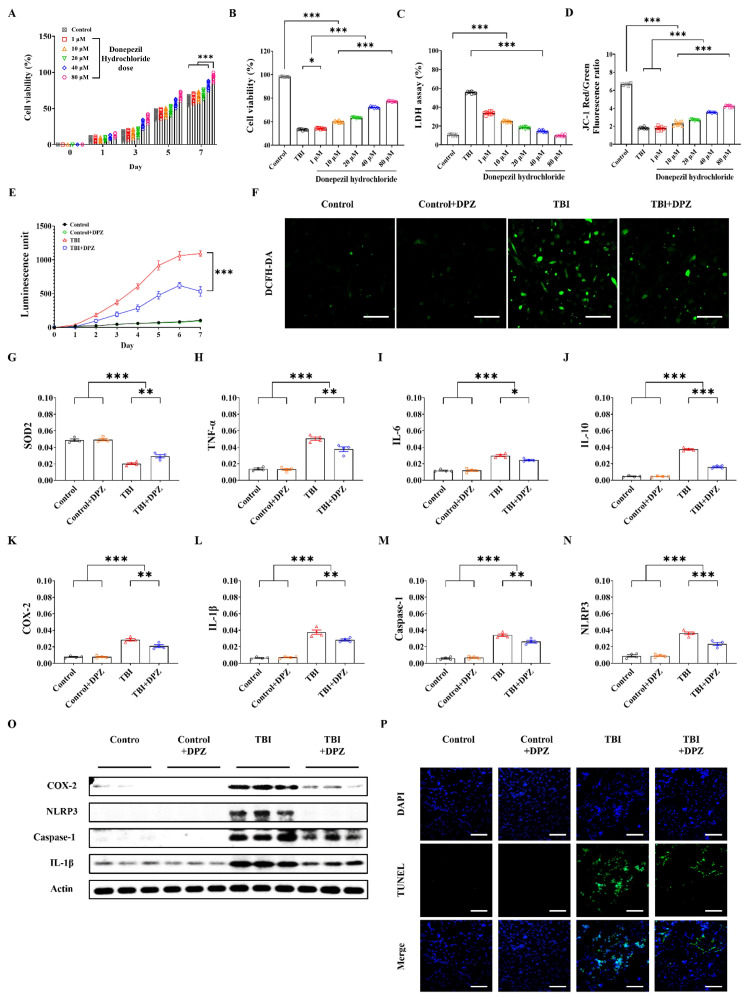
Results of the in vitro study. (**A**,**B**) Cell counting kit-8 (CCK-8) viability assay in SH-SY5Y cells with different dosage of donepezil (DPZ) and in in vitro traumatic brain injury model (n = 9, each group). (**C**–**F**) Comparison of lactate dehydrogenase (LDH) test (n = 9, each group), 5,5′,6,6′-tetrachloro-1,1′,3,3′-tetraethylbenzimi-dazolylcarbocyanine iodide (JC-1) (n = 9, each group), reactive oxygen species (ROS) scavenging (n = 6, each group), and 2′-7′-dichlorodihydrofluorescein diacetate (DCFH-DA) staining (n = 6 in each group). (**G**–**P**) Differences in mRNA expression levels, Western blotting (n = 4, each group), and terminal deoxynucleotidyl transferase dUTP nick end labeling (TUNEL)-positive cells (green, n = 6, each group) according to donepezil treatment. All in vitro experiments were repeated three times. Scale bar = 200 μm. Error bars indicate SEM. * *p* < 0.05, ** *p* < 0.01, and *** *p* < 0.005. Superoxide dismutase 2, SOD2; tumor necrosis factor, TNF-α; Interleukin-6, IL-6; Interleukin-10, IL-10; Cyclooxygenase-2, COX-2; NOD-like receptor protein 3, NLRP3; Caspase-1; Interleukin-1 beta, IL-1β.

An in vitro model of TBI in SH-SY5Y cells was introduced based on previous reports, including ours [20,21]. Cells were grown to 80–90% confluence in Ham’s F12/Minimum Essential Medium (MEM) (1:1) complete medium (Welgene, Gyeongsan, Republic of Korea) in six-well BioFlex culture plates (Flexcell International Corporation, McKeesport, PA, USA). An in vitro TBI model was generated using a Cell Injury Controller Ⅱ system (Flexcell International Corporation, McKeesport, PA, USA). The specific parameters considered were pulse duration (50 ms) and regulator pressure (3.5 PSI). The cells were incubated for 7 days following TBI. The cells were divided into three groups: control, i.e., cells without injury; TBI; and TBI with donepezil treatment. The optimal dosage of donepezil was chosen after testing various concentrations (e.g., 1, 10, 20, 40, and 80 μM; Figure 1A) [21].

### 2.2. In Vivo Model of TBI

Specific pathogen-free (SPF)-grade male C57BL/6J mice (seven to eight weeks of age) were used to generate an in vivo TBI model. Mice were divided into four groups: sham operation with saline treatment, sham operation with donepezil treatment, TBI with saline treatment, and TBI with donepezil treatment (18 mice in each group). Donepezil treatment was administered within 4 h post-TBI. A stereotaxic impactor (RWD-68099, RWD Life Science, Shenzhen, China) was used for TBI modeling while referencing a previous study [21]. Under 2.5% isoflurane anesthesia, the mice were positioned in a stereotaxic frame, and a 2 mm blunt tip was applied to the scalp while targeting specific parameters (M/L = −2.0 mm, A/P = −1.5 mm from the bregma; and depth = 2.0 mm) [21]. The impactor was set to a velocity of 3.5 m/s and a dwell time of 1.0 m/s. After induction of traumatic brain injury (TBI), anesthesia was discontinued. Donepezil was administered via intraperitoneal (IP) injection at a dose of 1 mg/kg/day for 7 days. To examine the histological analysis of mouse brain tissue, we performed transcardial perfusion with saline and 4% paraformaldehyde. Each experiment was repeated three times, and the results were evaluated in a blinded manner by three investigators (Supplementary Figures S1). GPower_3.1.9.7 software was used to determine the sample size of the data. The experimental procedures were approved by the Institutional Animal Care and Use Committee of the respective university (approval no. Hallym2020-51).

### 2.3. In Vitro Study

The CCK-8 assay (DAWINBIO, Hanam, Republic of Korea) was performed to identify the optimal dosage of donepezil. Dichloro-dihydro-fluorescein diacetate (DCFH-DA) and terminal deoxynucleotidyl transferase dUTP nick end-labeling (TUNEL) assay were conducted to assess reactive oxygen species (ROS) production and apoptotic cells, respectively [21,22,23,24]. Quantitative real-time polymerase chain reaction (qRT-PCR) and Western blotting were used to measure the expressions of mRNA and its related protein. Mitochondrial membrane potential was assessed using JC-1 staining (Figure 1) [21,25].

### 2.4. In Vivo Study

#### 2.4.1. H&E Staining, Brain Water Content, and Fluoro-Jade B Staining

The cryosections of mouse brain tissues (10 µm) were deparaffinized by xylene and ethanol (2 × xylene, 50% xylene: 50% EtOH, 100% ethanol, 100% ethanol, 95% ethanol, 90% ethanol, 80% ethanol, 70% ethanol, each 3 min) and stained using the hematoxylin–eosin (BBC biochemical, Seoul, Republic of Korea) method (hematoxylin for 1 min; eosin for 30 s). Then, sections were dehydrated in ethanol and xylene and sealed with synthetic resin. Damaged lesions were quantified using ImageJ™ software version 1.54f and calculated according to the following formula: corrected injury volume = [(contralateral hemispheric volume) − (injured hemispheric volume)]/[(contralateral hemispheric volume) × 2] × 100% [21,26]. Brain water content was assessed by dry/wet weight measurement. Brain water content was determined using the following formula: % water content = 100 × (wet weight − dry weight)/wet weight [21,22]. Brain tissues were coronally cryosectioned with a thickness of 30 μm [21]. The sectioned slides were immersed in 0.06% potassium permanganate for 15 min and washed for 2 min with distilled water. Slides were stained with 0.001% Fluoro-Jade B (FJB; Histo-Chem Inc., Jefferson, AR, USA) solution for 45 min. After washing two times in distilled water for 2 min, the slides were dried at room temperature at least overnight in the dark and cover-slipped with D.P.X. (Sigma-Aldrich Co., St. Louis, MI, USA). The stained brain tissues were observed using a fluorescence microscope under the wavelength range from 450 to 490 nm (Carl Zeiss, Oberkochen, Germany) [22]. ImageJ software was used to count the number of FJB-positive cells.

##### 2.4.2. qRT-PCR and Western Blotting

To compare the expression of mRNA and protein in each group, the samples were randomly divided two groups and used for qRT-PCR and Western blotting (WB). Only the cortex portion of the brain tissue was anatomically isolated and analyzed using qRT-PCR and WB. The quantification of the mRNA expression through qRT-PCR was calculated using the comparative threshold (ΔΔCt) method with β-actin. Total RNA in brain tissues and cells was isolated using the easy-BLUE kit (Invitrogen, Carlsbad, CA, USA). cDNA synthesis was performed using the Maxime Oligo RT PreMix kit (iNtRON Biotechnology, Seongnam, Republic of Korea) according to the manufacturer’s instructions. PCR reactions were carried out with a Rotor-Gene Q instrument (Qiagen) using the 2× Rotor-Gene SYBR Green PCR Master Mix (Qiagen, San Diego, CA, USA). PCR amplification consisted of 40 cycles, comprising denaturation at 94 °C for 15 s, annealing at 55 °C for 30 s, and extension at 70 °C for 30 s [21]. Western blotting was assessed based on the optical density of specific proteins relative to that of β-actin. Brain tissue and cells were lysed in RIPA buffer supplemented with proteinase inhibitor K. Protein concentrations were determined using the Pierce BCA Protein Assay Kit (Thermo Scientific, Waltham, MA, USA). Equal amounts of cell extracts were loaded for Western blotting analysis. Quantification through qRT-PCR Western blotting was assessed based on the optical density relative to that of β-actin. Supplementary Table S1 provides details about the primer sequences used in this process.

##### 2.4.3. Cognitive Function Test

Three tests were used to evaluate cognitive functions: the Morris water maze (MWM), the Y-maze, and novel object recognition (NOR) [21,22]. The method used for each test is detailed in the Supplementary Methods section. Cognitive function tests were performed every day for seven days. All data were recorded using a video tracking system and analyzed based on heat-map images (Noldus Ethovision, Leesburg, VA, USA). The red and blue colors, respectively, indicate frequently visited and less-visited areas. The results were reviewed in a blind manner.

##### 2.4.4. Statistical Analysis

Data were presented in the form of means ± standard errors of the mean (SEM). Student’s *t*-test and one-way ANOVA with post hoc Bonferroni correction were conducted to assess all possible pair-wise comparisons [21,23,27]. Baseline values were used as covariates for cognitive function tests in the statistical analysis section. The statistical significance levels of <0.05, 0.01, and 0.005 are denoted by *, **, and ***, respectively. GraphPad Prism software (v.8.02; GraphPad Software Inc., San Diego, CA, USA) was used for statistical analysis.

## 3. Results

### 3.1. Cytoprotective Effect in In Vitro TBI

After 24 h, the CCK-8 assay showed increases in both cell viability and donepezil concentration (Figure 1A). After donepezil treatment, there was an increase in cell viability in CCK-8 assay and a decrease in LDH levels, along with a restoration of the JC-1 level compared to the control or TBI-without-donepezil groups (Figure 1B–D). The treatment efficacy was proportional to the donepezil concentration, so 80 µM of donepezil was selected as the optimal treatment dosage. TBI treated with donepezil showed a significant reduction in ROS, as determined by DCFH-DA assay (Figure 1E,F). Moreover, the mRNA expressions of the neuroinflammation markers (e.g., SOD2, TNF-α, IL-6, IL-10, COX-2, IL-1β, Caspase-1, and NLRP3) and their Western blotting (e.g., COX-2, NLRP3, Caspase-1, and IL-1β) were decreased following donepezil treatment (Figure 1G–O and Supplementary Figure S3). Moreover, cellular apoptosis decreased after donepezil treatment, as seen on TUNEL staining (Figure 1P).

**Figure 2:**
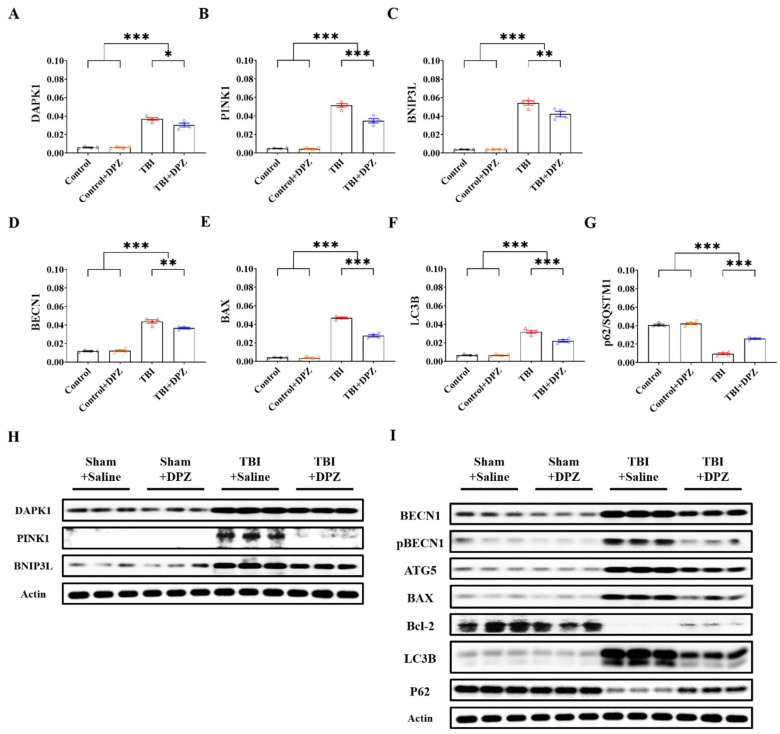
mRNA expression (n = 4, each group) (**A**–**G**) and Western blotting (n = 3, each group) (**H**,**I**) in autophagy- and mitophagy-related markers based on 80 μM donepezil (DZP) treatment in in vitro model of traumatic brain injury using SH-SY5Y. Error bars indicate SEM. * *p* < 0.05, ** *p* < 0.01, and *** *p* < 0.005. Death-associated protein kinase 1, DAPK1; PTEN-induced kinase 1, PINK1; BCL2/adenovirus E1B 19 kDa protein-interacting protein 3-like, BNIP3L; Beclin 1, BECN1; Bcl-2-associated X-protein, BAX; microtubule-associated proteins 1A/1B light-chain 3B (LC3B); Sequestosome 1; p62/SQSTM1; Actin, β-actin.

We evaluated mRNA expression and Western blotting of relevant markers associated with autophagy and mitophagy. The TBI-with-donepezil group exhibited significantly decreased levels of autophagy- and mitophagy-related markers compared to the TBI-without-donepezil group. Overall, donepezil decreased autophagy- and mitophagy-related markers after TBI (Figure 2). Detailed specific values and statistics are provided in Supplementary Tables S2 and S3, and Supplementary Figure S3.

### 3.2. Neuroprotective Effects in In Vivo TBI

**Figure 3:**
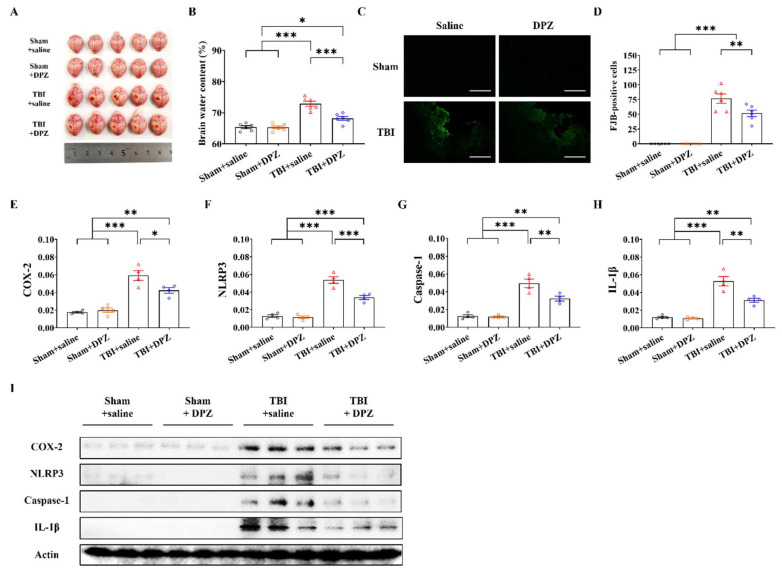
Results of the in vivo study using traumatic brain injury model. (**A**,**B**) Comparison of optical visible image of mouse brains (n = 5, each group) and brain water content (n = 6, each group). (**C**,**D**) Representative images of FJB-positive cells and their differences (n = 6, each group). Scale bar = 200 μm. (**E**–**I**) mRNA expression (n = 4, each group) and Western blotting of COX-2, NLRP3, Caspase-1, and IL-1β according to donepezil (DZP) treatment (n = 3, each group). Scale bar = 200 μm. Error bars indicate SEM. * *p* < 0.05, ** *p* < 0.01, and *** *p* < 0.005. Fluoro-jade, FJB; Cyclooxygenase-2, COX-2; NOD-like receptor protein 3, NLRP3; Caspase-1; Interleukin-1 beta, IL-1β.

In total, 72 mice were divided into four groups: sham operation (n = 18), sham operation with donepezil treatment (n = 18), TBI (n = 18), and TBI with donepezil treatment (n = 18). In each group, we collected samples to analyze RNA and protein (n = 6), FJB staining (n = 6), and brain water content (n = 6). TBI induced cortical tissue losses and brain swelling, the degrees of which were both shown to be alleviated after donepezil treatment (Figure 3A,B). FJB-positive cells in the cortex were also reduced after donepezil treatment (Figure 3C,D). TBI with donepezil showed significantly decreased mRNA and Western blotting expressions of neuroinflammation-related proteins, including COX-2, NLRP3, Caspase-1, and IL-1β (Figure 3E–I, Supplementary Figure S3, and Supplementary Tables S3 and S4).

**Figure 4:**
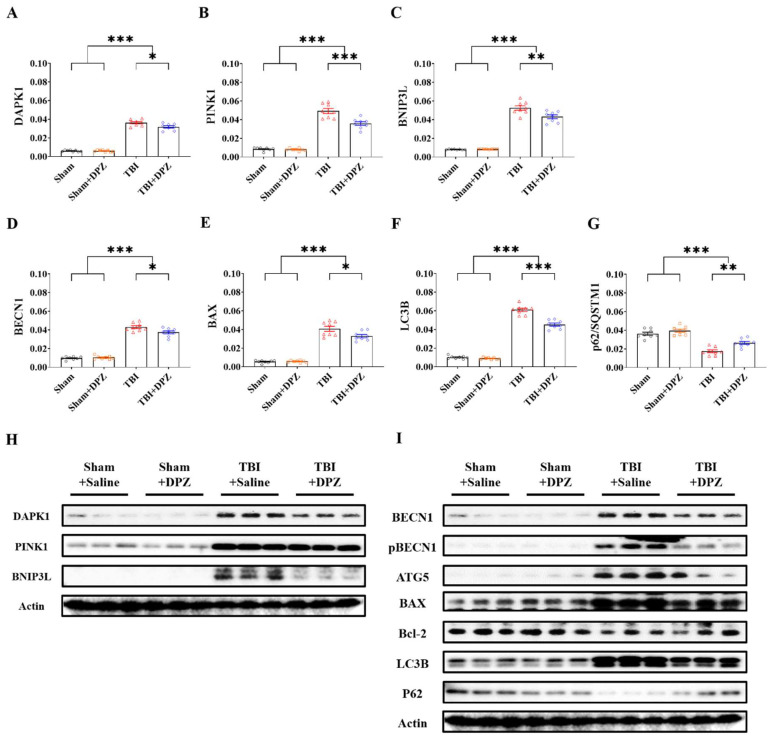
Differences in mRNA expression in (n = 8, each group) (**A**–**G**) and Western blotting (n = 3, each group) (**H**,**I**) in autophagy- and mitophagy-related markers based on donepezil (DZP) treatment in in vivo model of traumatic brain injury (TBI). Error bars indicate SEM. * *p* < 0.05, ** *p* < 0.01, and *** *p* < 0.005. Death-associated protein kinase 1, DAPK1; PTEN-induced kinase 1, PINK1; BCL2/adenovirus E1B 19 kDa protein-interacting protein 3-like, BNIP3L; Beclin 1, BECN1; Bcl-2-associated X-protein, BAX; microtubule-associated proteins 1A/1B light-chain 3B, LC3B; Sequestosome 1; p62/SQSTM1; Actin, β-actin.

TBI mice with donepezil treatment exhibited decreased expression levels of mRNA and Western blotting in both autophagy- and mitophagy-related markers compared to those without donepezil (Figure 4 and Supplementary Figure S3). Among the markers, BNIP3L exhibited the most statistical significance via Western blotting (Supplementary Table S5).

**Figure 5:**
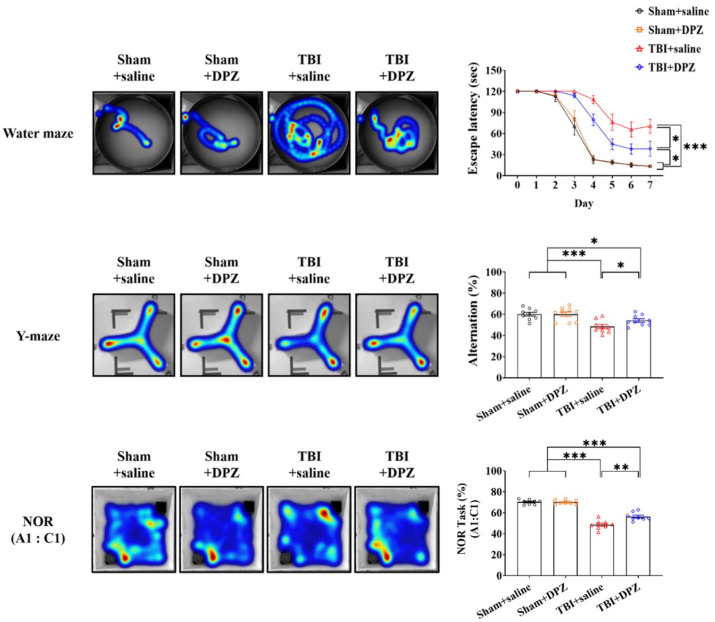
Cognitive function tests after donepezil (DZP) treatment in in vivo mouse model of traumatic brain injury (TBI) (n = 9, each group). The images are representative of the group. Error bars indicate SEM. * *p* < 0.05, ** *p* < 0.01, and *** *p* < 0.005. Novel object recognition, NOR. Red and blue indicate the highest or lowest colors in heatmaps.

TBI mice displayed a noticeable increase in escape latency over time in the water maze test, a lowered alteration rate in the Y-maze test, and lower preference index scores in the NOR test. After donepezil treatment, TBI mice exhibited improved cognitive performance, such as decreased escape latency in the water maze test and increased alteration rate in the Y-maze test, along with improved preference index scores for the new object (Figure 5 and Supplementary Table S4).

## 4. Discussion

Cognitive impairment following TBI is common, but there are still no appropriate drugs in clinical practice to ameliorate such a cognitive impairment occurring in TBI patients, other than that occurring after mild TBI. Unlike AD, the specific mechanism of TBI-driven cognitive impairment has yet to be widely investigated. Moreover, many related studies have focused on the similar association of pathological changes during AD when assessing TBI-related cognitive impairment. Aβ plaques can be detected within a few hours after TBI [28]. Rehman et al. [29] found that the inhibition of JNK led to reductions in amyloid precursor protein (APP) expression and Aβ production, with improved behavior and motor function after TBI. Johnson et al. [28] also reported that the imbalance between Aβ genesis and catabolism caused by TBI may result in rapid accumulations of both APP and Aβ plaque in damaged axons. Although these findings showed the pathogenic similarity of TBI-related cognitive impairment with AD, the cognitive impairment that occurs in the acute phase after TBI is likely to be caused by neuroinflammation or dysfunctional homeostasis. Therefore, we evaluated changes in neuroinflammation, as well as autophagy- and mitophagy-related markers after donepezil treatment.

Among various inflammation markers, the most significant difference between TBI mice with and without donepezil treatment was in the expression of COX-2. COX-2 has attracted attention for the role it plays in cognitive impairment through the interaction between neuroinflammation and neurodegeneration conditions in the pathogenesis of AD. COX-2 is expressed in pathological inflammatory conditions or normal physiologic conditions of synaptic plasticity [30,31]. Accordingly, increased COX-2 expression caused by TBI may activate microglia or astroglia, which, in turn, worsens inflammatory responses and increases microsomal PGE synthase (mPGES)-1 activity [31,32]. Guan et al. [33] reported that COX-2 was closely associated with both the propagation of Aβ and the reduction of glycosylation of tau in AD. The administration of a selective COX-2 inhibitor within 2 h after injury has been shown to lead to improved cognitive performance [34]. Moreover, the combination of donepezil with non-steroidal anti-inflammatory drugs (NSAIDs) has been shown to protect neuronal cell injury by β42 in vitro with alleviated inflammatory responses [35]. To this point, there have been few studies focusing on the effect of donepezil on cognitive function via COX-2 activity following TBI. ACh modulates COX-2 enzyme activity. Goschorska et al. [36] reported that ACh inhibitors such as donepezil and rivastigmine led to the decreased production of both COX-2 and PGE2 in macrophages. Accordingly, there is a need for further research scrutinizing the specific mechanism of the direct inflammation-relieving effect of donepezil or its indirect inflammation-relieving effect through AC regulation in early cognitive impairment after TBI.

Non-excitatory amino acids (NEAAs) play a crucial role in the pathogenesis of TBI. Specifically, NEAAs such as L-alanine, L-glutamine, glycine, L-histidine, L-serine, taurine, and L-threonine can accelerate synaptic transmission suppression and cause neuronal membrane depolarization during hypoxia, leading to neuronal damage [15]. These amino acids can adversely affect synaptic transmission depending on their concentration and combination, contributing to cognitive decline after TBI. System N and the alanine-serine-cysteine transporter 2 (ASCT2) are involved in the harmful effects of NEAAs during hypoxia, making them potential therapeutic targets for TBI treatment. Therefore, it is essential to explore the impact of donepezil on these NEAA-related pathways to mitigate early cognitive impairment post-TBI.

Autophagy and mitophagy have been proposed as a new target of therapeutic intervention for TBI [37]. In our study, BNIP3L showed the most statistically significant difference between TBI with and without donepezil treatment. BNIP3L damages BCL2-BECN1 complex and leads to autophagosome formation [38]. BNIP3L is also involved in mitophagy. Mitophagy largely proceeds through PTEN-induced putative kinase 1 (PINK1)/Parkin-mediated ubiquitin pathway and receptor-mediated pathway [39]. BNIP3L is known to be involved in mitochondrial receptor-mediated pathway through its binding with Atg8s like light chain 3 (LC3), which recruits autophagosomes [38]. The effects of autophagy and mitophagy are influenced by initial TBI severity. Increased autophagy flux may offer protection in mild TBI, but in severe TBI, the inhibition of autophagy flux may lead to neuronal injury [40]. Accordingly, appropriate augmentation or restoration of autophagy flux in brain cells or specific mitochondria may represent a potential therapeutic target for cognitive impairment following TBI [40]. In a study that did not focus on TBI, donepezil-treated rats with cardiac ischemia/reperfusion injury demonstrated improved left ventricular function, along with a smaller myocardial infarct size and arrhythmia compared to sham controls via cardiac mitochondrial protection [41]. Moreover, mitophagy dysfunction with reduced BNIP3L was observed in a murine model with a GBA mutant that is associated with Parkinson’s disease (PD) without changes in PARK2 and ubiquitin [38]. These findings suggested that the regulation of BNIP3L in autophagy and mitophagy could serve as a potential molecular target for drugs such as donepezil in TBI-related cognitive impairment.

TBI still represents a major burden worldwide due to its nature of high incidence, mortality, and morbidity. About 69 million patients are estimated to have TBI annually [42]. According to the World Health Organization, TBI accounts for approximately 30% of all injury-related mortality [43]. Elderly people are particularly vulnerable to TBI due to decreased physical function; in these cases, cognitive impairment becomes a major issue, along with functional recovery in neurology [44]. There are still no appropriate medications for cognitive impairment following TBI. Of course, it is meaningful to develop new drugs, but it is easier to consider expanding the indications for existing drugs, such as donepezil, which is already widely used in clinical practice. When using donepezil in TBI patients in clinical practice, there are two issues that are difficult to address based on the current research results: One is whether donepezil administered immediately after TBI helps recover cognitive impairment in the early phase in patients with more severe injuries than mild TBI. The other one is what the specific treatment mechanism is if it indeed helps in recovering early cognitive function after trauma. To address these issues, we investigated the potential effect of donepezil on cognition in a mouse model with moderate TBI and explored the treatment mechanism, while focusing on neuroinflammation, mitophagy, and autophagy, each for the first time. Nevertheless, our study has several limitations that require caution in interpretation. First, we focused on early TBI cognitive impairment following moderate TBI. While patients with mild TBI tend to experience a temporary decline in cognitive function that improves over time, patients with moderate TBI are more likely to have cognitive impairment [45]. Accordingly, we believe that the proper management of impaired cognitive function from the beginning may help prevent continued and worsening cognitive impairment. However, our study did not address the effect of donepezil on long-term cognition or the severe severity of TBI, which requires further validation in a future study. Second, we did not investigate hippocampal changes after TBI. Due to the nature of TBI, the most severely damaged regions could be the cortex, but hippocampal injury can also occur. It is still unclear to what extent neurogenesis occurring in the hippocampus after TBI contributes to cognitive impairment in the acute stage. Additional limitations might be considered, such as the use of only male mice, thus excluding the influence of estrogen; the potential influence of anesthesia on outcomes; and which mice can enter torpor, a hibernation-like state, after trauma which may impact their response to TBI. Therefore, the experimental results should be confirmed by correlative clinical findings on patients. Nevertheless, additional research should further investigate the effect of donepezil on neurogenesis in the hippocampus following moderate TBI. Moreover, based on our findings with additional results in in vivo experiments, the efficacy of donepezil on early cognitive impairment should be demonstrated in a relatively large number of moderate TBI patients.

## 5. Conclusions

Our findings suggest that donepezil, administered immediately after trauma, may be beneficial in improving cognitive impairment in the early period after moderate TBI via ameliorating neuroinflammation and autophagy and mitophagy.

## Data Availability

Data are available from the corresponding author upon reasonable request.

## Supplementary Materials

The following supporting information can be downloaded at: https://www.mdpi.com/article/10.3390/life14070839/s1, Figure S1. (A and B) Images of in vitro traumatic brain injury (TBI) modeling with a cell injury controller system and experimental design (C and D). Images of in vivo TBI modeling with controlled cortical impact and experimental design. DZP indicates donepezil; I.P, intraperitoneal; Tx, treatment. Figure S2. Details of Western blotting value comparison in in vitro (A) and in vivo models of traumatic brain injury (B). Figure S3. Original unprocessed images of Western blotting in in vitro (A) and in vivo (B) models of traumatic brain injury. Table S1. List of qRT-PCR primers used in this study. Table S2. Details of statistical significance levels regarding cell viability, LDH assay, JC-1, ROS scavenging activity, DCFH-DA, and TUNEL assay in in vitro TBI model. Table S3. Details of mRNA expressions in in vitro TBI model. Table S4. Details of statistical significance regarding brain water content, FJB staining, and cognition tests in in vivo TBI model. Table S5. Details of mRNA expressions in in vivo TBI models according to donepezil (DPZ) treatment. Table S6. Details of statistical significance in Western blotting in in vitro and in vivo TBI models. Supplemental methods. References [46,47] are cited in the supplementary materials.
