# Supplementary material for: Therapeutic Effect of Donepezil on Neuroinflammation and Cognitive Impairment after Moderate Traumatic Brain Injury
Source: Life (Basel). 2024 Jul 1;14(7):839. doi: 10.3390/life14070839 (PMC11278464; doi:10.3390/life14070839)
Supplement: Supplementary file 1 [file life-14-00839-s001.zip › life-3044269-supplementary.pdf]

Supplemental Data

Supplemental Figure S1. (A and B) Images of *in vitro* traumatic brain injury (TBI) modeling with a cell injury controller system and experimental design **(C and D)**. Images of *in vivo* TBI modeling with controlled cortical impact and experimental design. DZP indicates donepezil; I.P, intraperitoneal; Tx, treatment.

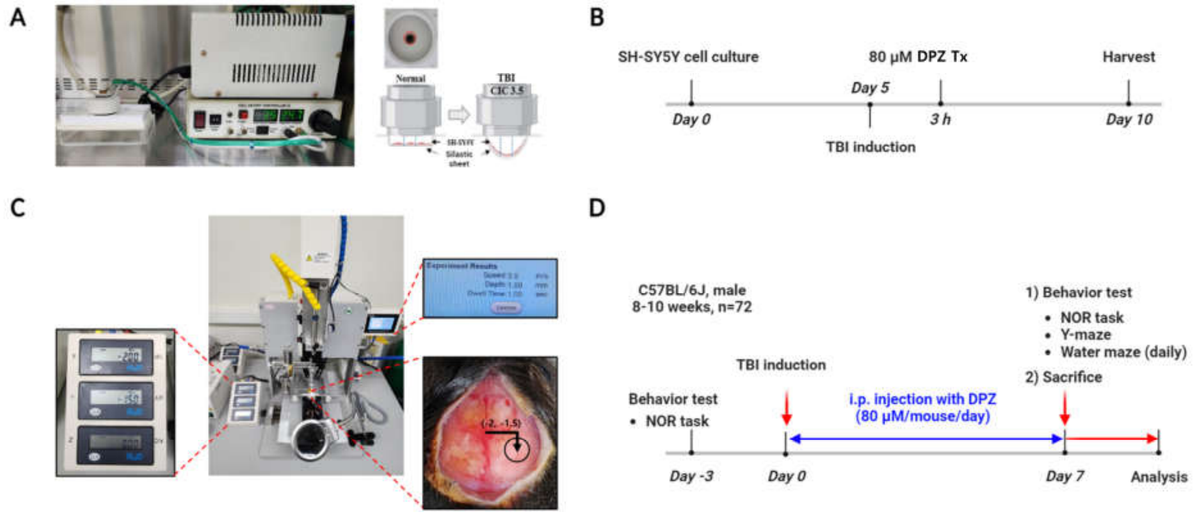

Supplemental Figure S2. Details of Western blotting value comparison in *in vitro* (A) and *in vivo* models of traumatic brain injury (B).

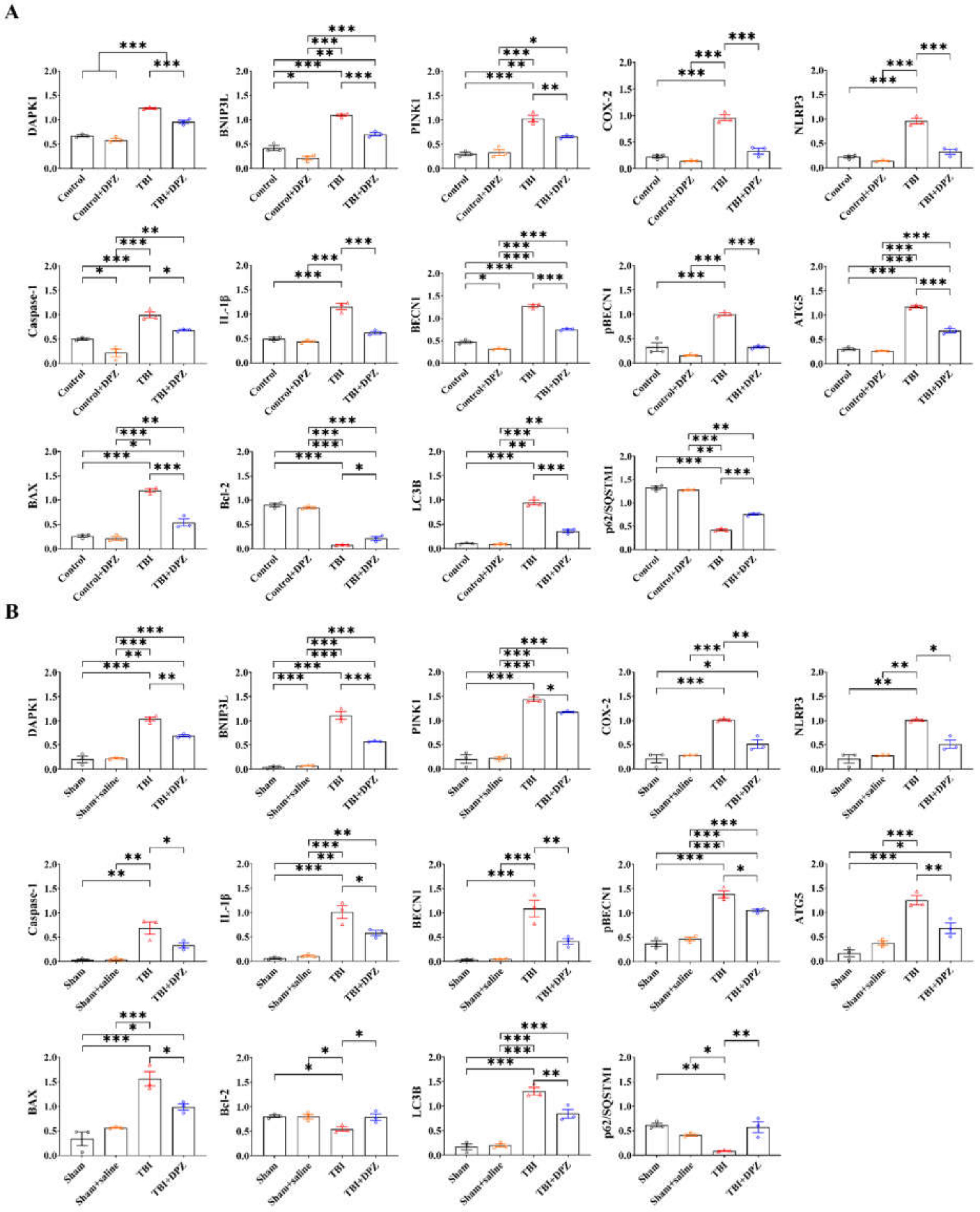

Supplemental Figure S3. Original unprocessed images of Western blotting in *in vitro* (A) and *in vivo* (B) models of traumatic brain injury.

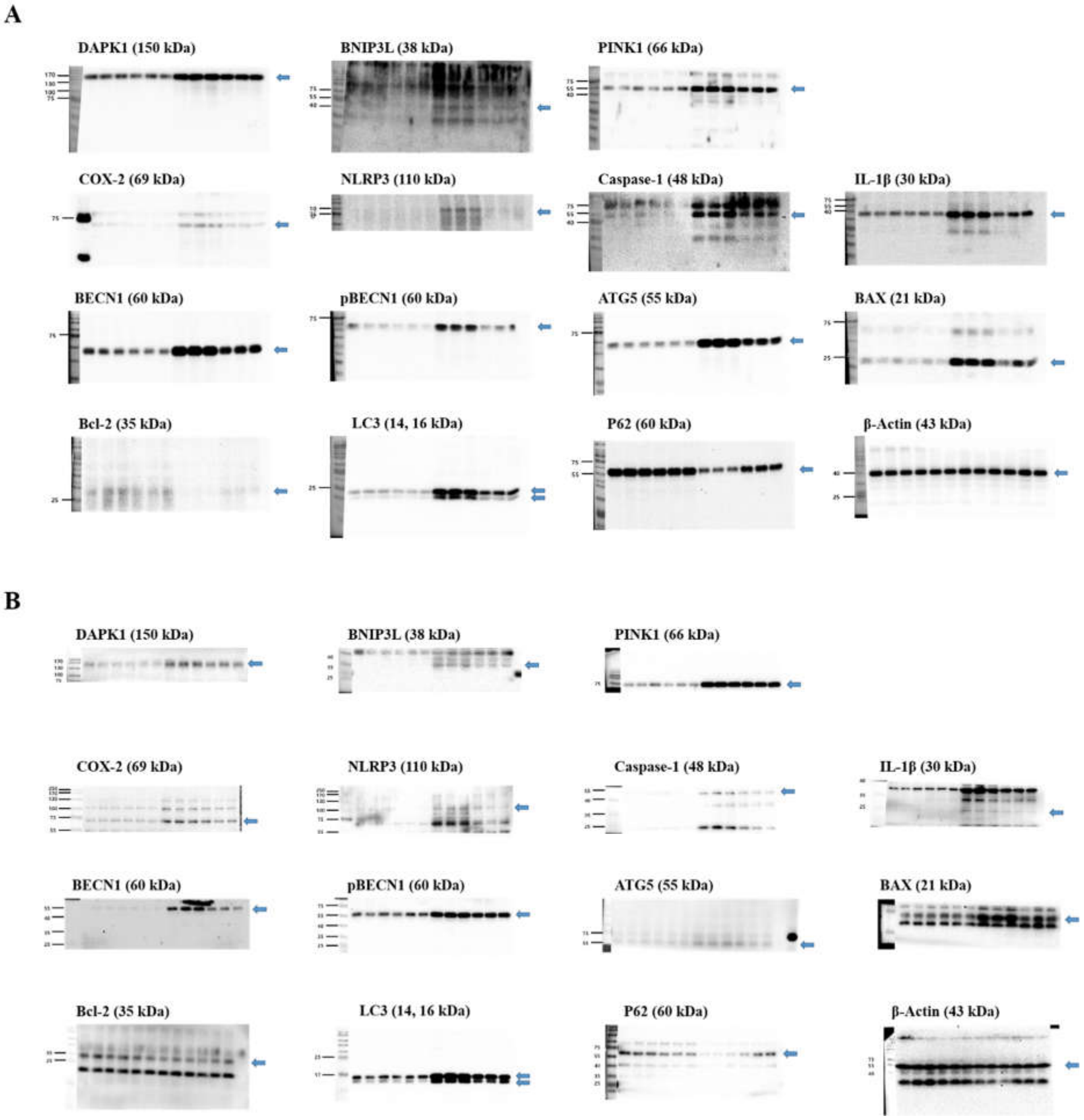

Supplemental Table S1. List of qRT-PCR primers used in this study.

		Primer sequence (5' -3')	
**Gene**	**Species**	**Forward**	**Reverse**
GAPDH	Mouse	5'-TGA CCT CAA CTA CAT GGT CTA CA-3'	5'-CTT CCC ATT CTC GGC CTT G-3'
	Human	5'-AAA ATC AAG TGG GGC GAT GC-3'	5'-AGG AGG CAT TGC TGA TGA TCT-3'
DAPK1	Mouse	5'-AGA TGT GGT CCG CTA CCT CTG T-3'	5'-ATG CTC GTG CTG TTC TGC CTT G-3'
	Human	5'-GAC CGT GAA GCA TTA CCT GAG-3'	5'-GCT GCT GAA GCT TTC CTT GTA-3'
PINK1	Mouse	5'-CGA CAA CAT CCT TGT GGA GTG G-3'	5'-CAT TGC CAC CAC GCT CTA CAC T-3'
	Human	5'-GTA TGA AGC CAC CAT GCC TAC-3'	5'-CAT CAT CTT GAT GGC CAA GGG TC-3'
BNIP3L	Mouse	5'-GCA TGA GGA AGA GTG GAG CCA T-3'	5'-AAG GTG TGC TCA GTC GTT TTC CA-3'
	Human	5'-ACA ACA ACA ACT GCG AGG AAA-3'	5'-GAG GAT GAG GAT GGT ACG TGT-3'
LC3B	Mouse	5'-GTC CTG GAC AAG ACC AAG TTC C-3'	5'-CCA TTC ACC AGG AGG AAG AAG G-3'
	Human	5'-GAG AAG CAG CTT CCT GTT CTG G-3'	5'-GTG TCC GTT CAC CAA CAG GAA G-3'
BECN1	Mouse	5'-CAG CCT CTG AAA CTG GAC ACG A-3'	5'-CTC TCC TGA GTT AGC CTC TTC C-3'
	Human	5'-ACC GTG TCA CCA TCC AGG AA-3'	5'-GAA GCT GTT GGC ACT TTC TGT-3'
BAX	Mouse	5'-AGG ATG CGT CCA CCA AGA AGC T-3'	5'-TCC GTG TCC ACG TCA GCA ATC A-3'
	Human	5'-TCA GGA TGC GTC CAC CAA GAA G-3'	5'-TGT GTC CAC GGC GGC AAT CAT C-3'
p62/SQSTMI	Mouse	5'-ACA CCT GCT TCT GGA GGA ACA G-3'	5'-TTG GAG GTG CTG CCA CTT GAG A-3'
	Human	5'-CTG GGA CTG AGA AGG CTC AC-3'	5'-GCA GCT GAT GGT TTG GAA AT-3'
SOD2	Mouse	5'-CCA AGA CCT GCC TTA CGA CTA-3'	5'-GGT GGC GTT GAG ATT GTT GA-3'
	Human	5'-GGA AGC CAT CAA ACG TGA CTT-3'	5'-GCA GTG GAT CCT GAT TTG GAC-3'
TNF-α	Mouse	5'-GCT GTC CCT GCG CTT CA-3'	5'-CTC GTC CCC AAT GAC ATC CT-3'
	Human	5'-TGT AGC CCA TGT TGT AGC AAA-3'	5'-GAG GAC CTG GGA GTA GAT GAG GTA-3'
IL-6	Mouse	5'-GGA TAC CAA CTA TTG CTT CAG CTC C-3'	5'-AGG CTC CAA ATA TAG GGG CAG GGT C-3'
	Human	5'-CCA CGG CCT TCC CTA CTT C-3'	5'-TTG GGA GTG GTA TCC TCT GTG A-3'
COX-2	Mouse	5'-GAT TGA CAG CCC ACC AAC TT-3'	5'-CGG GAT GAA CTC TCT CCT CA-3'
	Human	5'-CAT TCT TTG CCC AGC ACT TCA C-3'	5'-GAC CAG GCA CCA GAC CAA AGA C-3'
NLRP3	Mouse	5'-CGA GAC CTC TGG GAA AAA GCT-3'	5'-GCA TAC CAT AGA GGA ATG TGA TGT ACA-3'
	Human	5'-ACA GCC ACC TCA CTT CCA G-3'	5'-CCA ACC ACA ATC TCC GAA TG-3'
Caspase-1	Mouse	5'-GAT GGC ACA TTT CCA GGA CTG A-3'	5'-TGT TGC AGA TAA TGA GGG CAA GAC-3'
	Human	5'-GCA CAA GAC CTC TGA CAG CA-3'	5'-TTG GGC AGT TCT TGG TAT TC-3'
IL-1β	Mouse	5'-GAA ATG CCA CCT TTT GAC AGT G-3'	5'-TGG ATG CTC TCA TCA GGA CAG-3'
	Human	5'-GCC CTA AAC AGA TGA AGT GCT C-3'	5'-GAA CCA GCA TCT TCC TCA G-3'

Supplemental Table S2. Details of statistical significance levels regarding cell viability, LDH assay, JC-1, ROS scavenging activity, DCFH-DA, and TUNEL assay in *in vitro* TBI model.

	Cell viabililty - Normal with DPZ		
Condition	Group	Bonferroni's multiple comparisons test	
		Corrected *p*-value	Summary
Normal Day.7	Control vs. 1 µM	>0.9999	***
	Control vs. 10 µM	0.1357	***
	Control vs. 20 µM	0.0035	***
	Control vs. 40 µM	<0.0001	***
	Control vs. 80 µM	<0.0001	***
	1 µM vs. 10 µM	>0.9999	***
	1 µM vs. 20 µM	0.2859	*
	1 µM vs. 40 µM	<0.0001	***
	1 µM vs. 80 µM	<0.0001	***
	10 µM vs. 20 µM	>0.9999	***
	10 µM vs. 40 µM	<0.0001	***
	10 µM vs. 80 µM	<0.0001	***
	20 µM vs. 40 µM	<0.0001	***
	20 µM vs. 80 µM	<0.0001	***
	40 µM vs. 80 µM	0.0001	***

	Cell viabililty - TBI with DPZ		
Condition	Group	Bonferroni's multiple comparisons test	
		Corrected *p*-value	Summary
Normal	Control vs. TBI	<0.0001	***
	Control vs. 1 µM	<0.0001	***
	Control vs. 10 µM	<0.0001	***
	Control vs. 20 µM	<0.0001	***
	Control vs. 40 µM	<0.0001	***
	Control vs. 80 µM	<0.0001	***
TBI	TBI vs. 1 µM	0.0263	*
	TBI vs. 10 µM	<0.0001	***
	TBI vs. 20 µM	<0.0001	***
	TBI vs. 40 µM	<0.0001	***
	TBI vs. 80 µM	<0.0001	***
	1 µM vs. 10 µM	<0.0001	***
	1 µM vs. 20 µM	<0.0001	***
	1 µM vs. 40 µM	<0.0001	***
	1 µM vs. 80 µM	<0.0001	***
	10 µM vs. 20 µM	<0.0001	***
	10 µM vs. 40 µM	<0.0001	***
	10 µM vs. 80 µM	<0.0001	***
	20 µM vs. 40 µM	<0.0001	***
	20 µM vs. 80 µM	<0.0001	***
	40 µM vs. 80 µM	<0.0001	***

		LDH assay		JC-1	
Condition	Group	Bonferroni's multiple comparisons test		Bonferroni's multiple comparisons test	
		Corrected *p*-value	Summary	Corrected *p*-value	Summary
Normal	Control vs. TBI	<0.0001	***	<0.0001	***
	Control vs. 1 µM	<0.0001	***	<0.0001	***
	Control vs. 10 µM	<0.0001	***	<0.0001	***
	Control vs. 20 µM	<0.0001	***	<0.0001	***
	Control vs. 40 µM	<0.0001	***	<0.0001	***
	Control vs. 80 µM	<0.0001	***	<0.0001	***
TBI	TBI vs. 1 µM	>0.9999	ns	>0.9999	ns
	TBI vs. 10 µM	<0.0001	***	<0.0001	***
	TBI vs. 20 µM	<0.0001	***	<0.0001	***
	TBI vs. 40 µM	<0.0001	***	<0.0001	***
	TBI vs. 80 µM	<0.0001	***	<0.0001	***
	1 µM vs. 10 µM	<0.0001	***	<0.0001	***
	1 µM vs. 20 µM	<0.0001	***	<0.0001	***
	1 µM vs. 40 µM	<0.0001	***	<0.0001	***
	1 µM vs. 80 µM	<0.0001	***	<0.0001	***
	10 µM vs. 20 µM	<0.0001	***	<0.0001	***
	10 µM vs. 40 µM	<0.0001	***	<0.0001	***
	10 µM vs. 80 µM	<0.0001	***	<0.0001	***
	20 µM vs. 40 µM	<0.0001	***	<0.0001	***
	20 µM vs. 80 µM	<0.0001	***	<0.0001	***
	40 µM vs. 80 µM	<0.0001	***	<0.0001	***

	ROS scavenging activity		DCFH-DA		TUNEL	
Group	Bonferroni's multiple comparisons test		Bonferroni's multiple comparisons test		Bonferroni's multiple comparisons test	
	Corrected *p*-value	Summary	Corrected *p*-value	Summary	Corrected *p*-value	Summary
Control vs. Control+DPZ	>0.9999	ns	>0.9999	ns	>0.9999	ns
Control vs. TBI	<0.0001	***	<0.0001	***	<0.0001	***
Control vs. TBI+DPZ	<0.0001	***	<0.0001	***	<0.0001	***
Control+DPZ vs. TBI	<0.0001	***	<0.0001	***	<0.0001	***
Control+DPZ vs. TBI+DPZ	<0.0001	***	<0.0001	***	<0.0001	***
TBI vs. TBI+DPZ	<0.0001	***	<0.0001	***	<0.0001	***

Supplemental Table S3. Details of mRNA expressions in *in vitro* TBI model.

Neuroinflammation mRNA expression level

mRNA target	Group	Bonferroni's multiple comparisons test	
		Corrected *p*-value	Summary
SOD2	Control vs. Control+DPZ	>0.9999	ns
	Control vs. TBI	<0.0001	***
	Control vs. TBI+DPZ	<0.0001	***
	Control+DPZ vs. TBI	<0.0001	***
	Control+DPZ vs. TBI+DPZ	<0.0001	***
	TBI vs. TBI+DPZ	0.007	**
TNF-α	Control vs. Control+DPZ	>0.9999	ns
	Control vs. TBI	<0.0001	***
	Control vs. TBI+DPZ	<0.0001	***
	Control+DPZ vs. TBI	<0.0001	***
	Control+DPZ vs. TBI+DPZ	<0.0001	***
	TBI vs. TBI+DPZ	0.0031	**
IL-6	Control vs. Control+DPZ	>0.9999	ns
	Control vs. TBI	<0.0001	***
	Control vs. TBI+DPZ	<0.0001	***
	Control+DPZ vs. TBI	<0.0001	***
	Control+DPZ vs. TBI+DPZ	<0.0001	***
	TBI vs. TBI+DPZ	0.015	*
COX-2	Control vs. Control+DPZ	>0.9999	ns
	Control vs. TBI	<0.0001	***
	Control vs. TBI+DPZ	<0.0001	***
	Control+DPZ vs. TBI	<0.0001	***
	Control+DPZ vs. TBI+DPZ	<0.0001	***
	TBI vs. TBI+DPZ	0.0051	**
NLRP3	Control vs. Control+DPZ	>0.9999	ns
	Control vs. TBI	<0.0001	***
	Control vs. TBI+DPZ	0.0002	***
	Control+DPZ vs. TBI	<0.0001	***
	Control+DPZ vs. TBI+DPZ	0.0002	***
	TBI vs. TBI+DPZ	0.0004	***
Caspase-1	Control vs. Control+DPZ	>0.9999	ns
	Control vs. TBI	<0.0001	***
	Control vs. TBI+DPZ	<0.0001	***
	Control+DPZ vs. TBI	<0.0001	***
	Control+DPZ vs. TBI+DPZ	<0.0001	***
	TBI vs. TBI+DPZ	0.0053	**
IL-1ß	Control vs. Control+DPZ	>0.9999	ns
	Control vs. TBI	<0.0001	***
	Control vs. TBI+DPZ	<0.0001	***
	Control+DPZ vs. TBI	<0.0001	***
	Control+DPZ vs. TBI+DPZ	<0.0001	***
	TBI vs. TBI+DPZ	0.0057	**

Autophagy and Mitophagy mRNA expression level

mRNA target	Group	Bonferroni's multiple comparisons test	
		Corrected *p*-value	Summary
DAPK1	Control vs. Control+DPZ	>0.99	ns
	Control vs. TBI	<0.001	***
	Control vs. TBI+DPZ	<0.001	***
	Control+DPZ vs. TBI	<0.001	***
	Control+DPZ vs. TBI+DPZ	<0.001	***
	TBI vs. TBI+DPZ	0.02	*
PINK1	Control vs. Control+DPZ	>0.99	ns
	Control vs. TBI	<0.001	***
	Control vs. TBI+DPZ	<0.001	***
	Control+DPZ vs. TBI	<0.001	***
	Control+DPZ vs. TBI+DPZ	<0.001	***
	TBI vs. TBI+DPZ	<0.001	***
BNIP3L	Control vs. Control+DPZ	>0.99	ns
	Control vs. TBI	<0.001	***
	Control vs. TBI+DPZ	<0.001	***
	Control+DPZ vs. TBI	<0.001	***
	Control+DPZ vs. TBI+DPZ	<0.001	***
	TBI vs. TBI+DPZ	0.004	**
BECN1	Control vs. Control+DPZ	>0.99	ns
	Control vs. TBI	<0.001	***
	Control vs. TBI+DPZ	<0.001	***
	Control+DPZ vs. TBI	<0.001	***
	Control+DPZ vs. TBI+DPZ	<0.001	***
	TBI vs. TBI+DPZ	0.006	**
BAX	Control vs. Control+DPZ	>0.99	ns
	Control vs. TBI	<0.001	***
	Control vs. TBI+DPZ	<0.001	***
	Control+DPZ vs. TBI	<0.001	***
	Control+DPZ vs. TBI+DPZ	<0.001	***
	TBI vs. TBI+DPZ	<0.001	***
LC3B	Control vs. Control+DPZ	>0.99	ns
	Control vs. TBI	<0.001	***
	Control vs. TBI+DPZ	<0.001	***
	Control+DPZ vs. TBI	<0.001	***
	Control+DPZ vs. TBI+DPZ	<0.001	***
	TBI vs. TBI+DPZ	<0.001	***
p62/SQSTM1	Control vs. Control+DPZ	>0.99	ns
	Control vs. TBI	<0.001	***
	Control vs. TBI+DPZ	<0.001	***
	Control+DPZ vs. TBI	<0.001	***
	Control+DPZ vs. TBI+DPZ	<0.001	***
	TBI vs. TBI+DPZ	<0.001	***

Supplemental Table S4. Details of statistical significance regarding brain water content, FJB staining, and cognition tests in *in vivo* TBI model.

Neuroprotection assays

Test	Group	Bonferroni's multiple comparisons test	
		Corrected *p*-value	Summary
Brain water content	Sham+saline vs. Sham+DPZ	>0.9999	ns
	Sham+saline vs. TBI+saline	<0.0001	***
	Sham+saline vs. TBI+DPZ	<0.0001	***
	Sham+DPZ vs. TBI+saline	<0.0001	***
	Sham+DPZ vs. TBI+DPZ	<0.0001	***
	TBI+saline vs. TBI+DPZ	0.00740	**
FJB staining	Sham+saline vs. Sham+DPZ	>0.9999	ns
	Sham+saline vs. TBI+saline	<0.0001	***
	Sham+saline vs. TBI+DPZ	<0.0001	***
	Sham+DPZ vs. TBI+saline	<0.0001	***
	Sham+DPZ vs. TBI+DPZ	<0.0001	***
	TBI+saline vs. TBI+DPZ	1.10E-03	**

Cognition test

Test	Group	Bonferroni's multiple comparisons test	
		Corrected *p*-value	Summary
Y-maze	Sham+saline vs. Sham+DPZ	>0.9999	ns
	Sham+saline vs. TBI+saline	<0.0001	***
	Sham+saline vs. TBI+DPZ	4.08E-02	*
	Sham+DPZ vs. TBI+saline	<0.0001	***
	Sham+DPZ vs. TBI+DPZ	0.04630	*
	TBI+saline vs. TBI+DPZ	3.92E-02	*
Water maze	Sham+saline vs. Sham+DPZ	>0.9999	ns
	Sham+saline vs. TBI+saline	<0.0001	***
	Sham+saline vs. TBI+DPZ	0.04600	*
	Sham+DPZ vs. TBI+saline	<0.0001	***
	Sham+DPZ vs. TBI+DPZ	0.04430	*
	TBI+saline vs. TBI+DPZ	0.00730	**
NOR	Sham+saline vs. Sham+DPZ	>0.9999	ns
	Sham+saline vs. TBI+saline	<0.0001	***
	Sham+saline vs. TBI+DPZ	<0.0001	***
	Sham+DPZ vs. TBI+saline	<0.0001	***
	Sham+DPZ vs. TBI+DPZ	<0.0001	***
	TBI+saline vs. TBI+DPZ	0.00040	***

Supplemental Table S5. Details of mRNA expressions in *in vivo* TBI models according to donepezil (DPZ) treatment.

Neuroinflammation mRNA expression level

mRNA target	Group	Bonferroni's multiple comparisons test	
		Corrected *p*-value	Summary
SOD2	Control vs. Control+DPZ	>0.9999	ns
	Control vs. TBI	<0.0001	***
	Control vs. TBI+DPZ	<0.0001	***
	Control+DPZ vs. TBI	<0.0001	***
	Control+DPZ vs. TBI+DPZ	<0.0001	***
	TBI vs. TBI+DPZ	0.0122	*
TNF-α	Control vs. Control+DPZ	0.9999	ns
	Control vs. TBI	<0.0001	***
	Control vs. TBI+DPZ	<0.0001	***
	Control+DPZ vs. TBI	<0.0001	***
	Control+DPZ vs. TBI+DPZ	<0.0001	***
	TBI vs. TBI+DPZ	0.0015	**
IL-6	Control vs. Control+DPZ	>0.9999	ns
	Control vs. TBI	<0.0001	***
	Control vs. TBI+DPZ	<0.0001	***
	Control+DPZ vs. TBI	<0.0001	***
	Control+DPZ vs. TBI+DPZ	<0.0001	***
	TBI vs. TBI+DPZ	0.0206	*
COX-2	Control vs. Control+DPZ	>0.9999	ns
	Control vs. TBI	<0.0001	***
	Control vs. TBI+DPZ	<0.0001	***
	Control+DPZ vs. TBI	<0.0001	***
	Control+DPZ vs. TBI+DPZ	<0.0001	***
	TBI vs. TBI+DPZ	0.0028	**
NLRP3	Control vs. Control+DPZ	>0.9999	ns
	Control vs. TBI	<0.0001	***
	Control vs. TBI+DPZ	<0.0001	***
	Control+DPZ vs. TBI	<0.0001	***
	Control+DPZ vs. TBI+DPZ	<0.0001	***
	TBI vs. TBI+DPZ	0.0008	***
Caspase-1	Control vs. Control+DPZ	>0.9999	ns
	Control vs. TBI	<0.0001	***
	Control vs. TBI+DPZ	<0.0001	***
	Control+DPZ vs. TBI	<0.0001	***
	Control+DPZ vs. TBI+DPZ	<0.0001	***
	TBI vs. TBI+DPZ	0.0043	**
IL-1β	Control vs. Control+DPZ	>0.9999	ns
	Control vs. TBI	<0.0001	***
	Control vs. TBI+DPZ	0.0002	***
	Control+DPZ vs. TBI	<0.0001	***
	Control+DPZ vs. TBI+DPZ	0.0005	***
	TBI vs. TBI+DPZ	0.0001	***

Autophagy and Mitophagy mRNA expression level

mRNA target	Group	Bonferroni's multiple comparisons test	
		Corrected *p*-value	Summary
DAPK1	Control vs. Control+DPZ	>0.9999	ns
	Control vs. TBI	<0.0001	***
	Control vs. TBI+DPZ	<0.0001	***
	Control+DPZ vs. TBI	<0.0001	***
	Control+DPZ vs. TBI+DPZ	<0.0001	***
	TBI vs. TBI+DPZ	0.0101	*
PINK1	Control vs. Control+DPZ	>0.9999	ns
	Control vs. TBI	<0.0001	***
	Control vs. TBI+DPZ	<0.0001	***
	Control+DPZ vs. TBI	<0.0001	***
	Control+DPZ vs. TBI+DPZ	<0.0001	***
	TBI vs. TBI+DPZ	<0.0001	***
BNIP3L	Control vs. Control+DPZ	>0.9999	ns
	Control vs. TBI	<0.0001	***
	Control vs. TBI+DPZ	<0.0001	***
	Control+DPZ vs. TBI	<0.0001	***
	Control+DPZ vs. TBI+DPZ	<0.0001	***
	TBI vs. TBI+DPZ	0.0013	**
BECN1	Control vs. Control+DPZ	>0.9999	ns
	Control vs. TBI	<0.0001	***
	Control vs. TBI+DPZ	<0.0001	***
	Control+DPZ vs. TBI	<0.0001	***
	Control+DPZ vs. TBI+DPZ	<0.0001	***
	TBI vs. TBI+DPZ	0.0123	*
BAX	Control vs. Control+DPZ	>0.9999	ns
	Control vs. TBI	<0.0001	***
	Control vs. TBI+DPZ	<0.0001	***
	Control+DPZ vs. TBI	<0.0001	***
	Control+DPZ vs. TBI+DPZ	<0.0001	***
	TBI vs. TBI+DPZ	0.0125	*
LC3B	Control vs. Control+DPZ	>0.9999	ns
	Control vs. TBI	<0.0001	***
	Control vs. TBI+DPZ	<0.0001	***
	Control+DPZ vs. TBI	<0.0001	***
	Control+DPZ vs. TBI+DPZ	<0.0001	***
	TBI vs. TBI+DPZ	<0.0001	***
p62/SQSTM1	Control vs. Control+DPZ	0.9787	ns
	Control vs. TBI	<0.0001	***
	Control vs. TBI+DPZ	0.0011	**
	Control+DPZ vs. TBI	<0.0001	***
	Control+DPZ vs. TBI+DPZ	<0.0001	***
	TBI vs. TBI+DPZ	0.0025	**

Supplemental Table S6. Details of statistical significance in Western blotting in *in vitro* and *in vivo* TBI models.

	Group	Control vs. Control+DPZ		Control vs. TBI		Control vs. TBI+DPZ		Control+DPZ vs. TBI		Control+DPZ vs. TBI+DPZ		TBI vs. TBI+DPZ	
	Bonferroni's multiple comparisons test	Corrected p-value	Summary	Corrected p-value	Summary	Corrected p-value	Summary	Corrected p-value	Summary	Corrected p-value	Summary	Corrected p-value	Summary
	DAPK1	0.41	ns	<0.001	***	<0.001	***	<0.001	***	<0.001	***	<0.001	***
	BNIP3L	0.03	*	<0.001	***	0.004	**	<0.001	***	<0.001	***	<0.001	***
	PINK1	>0.99	ns	<0.001	***	0.006	**	<0.001	***	0.01	*	0.004	**
	COX-2	>0.99	ns	<0.001	***	0.65	ns	<0.001	***	0.07	ns	<0.001	***
	NLRP3	>0.99	ns	<0.001	***	>0.99	ns	<0.001	***	>0.99	ns	<0.001	***
	Caspase-1	0.03	*	<0.001	***	0.25	ns	<0.001	***	0.001	**	0.02	*
In vitro	IL-1β	>0.99	ns	<0.001	***	0.28	ns	<0.001	***	0.05	ns	<0.001	***
	BECN1	0.01	*	<0.001	***	<0.001	***	<0.001	***	<0.001	***	<0.001	***
	pBECN1	0.26	ns	<0.001	***	>0.99	ns	<0.001	***	0.26	ns	<0.001	***
	ATG5	>0.99	ns	<0.001	***	<0.001	***	<0.001	***	<0.001	***	<0.001	***
	BAX	>0.99	ns	<0.001	***	0.02	*	<0.001	***	0.007	**	<0.001	***
	Bcl-2	>0.99	ns	<0.001	***	<0.001	***	<0.001	***	<0.001	***	0.04	*
	LC3B	>0.99	ns	<0.001	***	0.002	**	<0.001	***	0.001	**	<0.001	***
	p62	>0.99	ns	<0.001	***	<0.001	***	<0.001	***	<0.001	***	<0.001	***

	Group	Sham vs. Sham+DPZ		Sham vs. TBI		Sham vs. TBI+DPZ		Sham+DPZ vs. TBI		Sham+DPZ vs. TBI+DPZ		TBI vs. TBI+DPZ	
	Bonferroni's multiple comparisons test	Corrected p-value	Summary	Corrected p-value	Summary	Corrected p-value	Summary	Corrected p-value	Summary	Corrected p-value	Summary	Corrected p-value	Summary
	DAPK1	>0.99	ns	<0.001	***	<0.001	***	<0.001	***	<0.001	***	0.002	**
	BNIP3L	>0.99	ns	<0.001	***	<0.001	***	<0.001	***	<0.001	***	<0.001	***
	PINK1	>0.99	ns	<0.001	***	<0.001	***	<0.001	***	<0.001	***	0.04	*
	COX-2	>0.99	ns	<0.001	***	0.04	*	<0.001	***	0.16	ns	0.002	**
	NLRP3	>0.99	ns	0.001	**	0.15	ns	0.001	**	0.12	ns	0.04	*
	Caspase-1	>0.99	ns	0.001	**	0.1	ns	0.001	**	0.12	ns	0.04	*
In vivo	IL-1β	>0.99	ns	<0.001	***	0.005	**	<0.001	***	0.01	**	0.02	*
	BECN1	>0.99	ns	<0.001	***	0.11	ns	<0.001	***	0.13	ns	0.005	**
	pBECN1	>0.99	ns	<0.001	***	<0.001	***	<0.001	***	<0.001	***	0.01	*
	ATG5	0.61	ns	<0.001	***	0.01	*	<0.001	***	0.18	ns	0.007	**
	BAX	>0.99	ns	<0.001	***	0.01	*	<0.001	***	0.13	ns	0.03	*
	Bcl-2	>0.99	ns	0.03	*	>0.99	ns	0.03	*	>0.99	ns	0.04	*
	LC3B	>0.99	ns	<0.001	***	<0.001	***	<0.001	***	<0.001	***	0.008	**
	p62	0.3	ns	0.002	**	>0.99	ns	0.03	*	0.62	ns	0.003	**

Supplemental Methods

Cognitive function tests

The Morris water maze (MWM) test was performed as described below: Before TBI, mice underwent daily 2-minute MWM trials for five consecutive days to learn and remember the location of a hidden platform (a clear column, 8 cm in diameter, 23.5 cm high) that was submerged in a round pool (100 cm in diameter, 45 cm deep) filled with water at a temperature between 21 – 23 °C. Seven days after TBI, mice performed a 2-minute MWM test. We recorded the time each mouse took to find the platform. These tests were conducted to assess the memory and cognitive abilities of the mice without any plagiarism [1].

Regarding the Y-maze test, mice were placed at the end of one arm of three symmetrically Y-shaped branches (length 40 cm, width 10 cm, height 12 cm) 7 days after TBI. After they explored the maze for 5 min, we counted the number of times they entered each arm. Each entry counted as 1 point. Their spontaneous alternation behavior was estimated using the following formula: (sequence of entries) / (total number of arms entered minus 2) × 100 [2].

The NOR test was performed according to the following sequences: Mice were adapted in an open field arena (dimensions: 45 cm length × 45 cm width × 45 cm height) for 10 min each day over two days. Over three days, the mice were given two identical objects named A1 and A2 in the same box for 10 min each day. Seven days after TBI induction, mouse activity was assessed by measuring travel distances in the box for 10 min while interacting with either a familiar object (A1) or a new object (C1). Interaction was defined as the mice standing on their hind legs near the object, sniffing it, or touching it with their nose. The preference object was regarded to be within 2 cm of mice.

References

1. Patil, S.S.; Sunyer, B.; Höger, H.; Lubec, G. Evaluation of spatial memory of C57BL/6J and CD1 mice in the Barnes maze, the Multiple T-maze and in the Morris water maze. *Behav. Brain Res.* **2009**, *198*, 58-68.
2. Baratz, R.; Tweedie, D.; Wang, J.Y.; Rubovitch, V.; Luo, W.; Hoffer, B.J.; Greig, N.H.; Pick, C.G. Transiently lowering tumor necrosis factor-α synthesis ameliorates neuronal cell loss and cognitive impairments induced by minimal traumatic brain injury in mice. *J. Neuroinflammation*. **2015**, *12*, 45.
